# Cartilage Resection in the Surgical Management of Ear Melanoma

**DOI:** 10.1245/s10434-025-17294-w

**Published:** 2025-04-28

**Authors:** Elan Novis, Joyce Tan, Danielle Vignati, Terence Wong, Robert V. Rawson, Jonathan R. Stretch, Serigne N. Lo, Thomas E. Pennington, Sydney Ch’ng, Kerwin F. Shannon, Andrew J. Spillane, Omgo E. Nieweg, John F. Thompson, Michael Rtshiladze, Richard A. Scolyer, Robyn P. M. Saw

**Affiliations:** 1 Melanoma Institute Australia, The University of Sydney, Sydney, NSW Australia; 2 Royal Prince Alfred Hospital, Sydney, NSW Australia; 3 The University of NSW, Sydney, NSW Australia; 4 Faculty of Medicine and Health, The University of Sydney, Sydney, NSW Australia; 5 NSW Health Pathology, Newcastle, NSW Australia; 6 Royal North Shore Hospital, Sydney, NSW Australia; 7 Charles Perkins Centre, The University of Sydney, Sydney, NSW Australia; 8 Mater Hospital, Wollstonecraft, NSW Australia

## Abstract

**Background:**

Melanoma of the ear accounts for approximately 1% of cutaneous melanomas. Management recommendations are based on small retrospective series and case reports. Resection of melanoma of the ear requires a delicate balance between disease clearance, preservation of function, and aesthetics. The role of cartilage resection in the wide excision of melanoma of the ear remains unclear. We aimed to compare outcomes in patients having wide excision of ear melanoma who had cartilage resected with those who had a cartilage-sparing approach.

**Methods:**

Data were obtained from the Melanoma Institute Australia (MIA) prospectively maintained database. All patients diagnosed with invasive melanoma involving the ear between 1990 and 2022 were included. Data analysis was performed to assess the association between cartilage resection and recurrence-free survival (RFS), melanoma-specific survival (MSS), and overall survival (OS).

**Results:**

Overall, 411 patients were included in the study, of whom 330 (80%) had cartilage resected and 81 (20%) had a cartilage-sparing resection. The cartilage resection group had a higher mean Breslow thickness (1.9 vs. 1.4 mm; *p* = 0.0002), whereas the cartilage-sparing group had a higher proportion of stage IA disease (60.5 vs. 39.7%; *p* = 0.041). Five (1.2%) patients had melanoma invading into perichondrium but not deeper. Cartilage resection had no impact on RFS {hazard ratio [HR] 0.82 (0.52–1.29); *p* = 0.39} or MSS (HR 0.89 (0.30–2.62); *p* = 0.83).

**Conclusion:**

The decision to resect cartilage as part of the wide excision of invasive ear melanoma should be tailored to the needs of the individual patient, however a cartilage-sparing approach does not appear to compromise MSS outcomes, particularly in early-stage disease.

Melanoma of the external ear accounts for approximately 1% of cutaneous melanoma cases and 7–14% of melanomas arising in the head and neck region.^1,2^ Current evidence for the surgical management of this subgroup of melanoma patients is limited to small retrospective case series and case reports.^3–13^ Additionally, few studies have compared melanoma-specific outcomes for patients who have and have not had cartilage-sparing resections, which is an important consideration in the decision process for resection and reconstruction of this anatomically complex body region. Some studies have suggested a worse prognosis and more aggressive growth pattern for ear melanomas and therefore advocated for more aggressive resections, including total or partial amputation.^13–17^ More recently, others have suggested a more conservative surgical approach without compromising oncological outcomes.^4–9^ In addition, current guidelines are based on studies that are poorly represented by ear melanomas and do not provide guidance on the required deep margin.^18–21^ There is therefore a need for further investigation of these patients to determine optimal margins, in particular the need to include cartilage in the resection if not clinically involved.

The external ear is a surgically complex body region, where oncological clearance must be balanced with good cosmetic outcome and maintenance of function, including the funneling of sound into the external acoustic meatus to facilitate hearing, and allowing placement of glasses and hearing aids if required. Preservation of cartilage may help to maintain these important functions. The relationship of the auricular cartilage to the invasion patterns of melanoma, recurrence, and survival outcomes has not yet been established.

This study aimed to compare melanoma-specific outcomes with either cartilage resection or cartilage-sparing management in patients with invasive melanoma of the external ear and to determine the frequency of invasion of melanoma into cartilage in these patients.

## Materials and Methods

Data were obtained from the Melanoma Institute Australia (MIA) prospectively maintained database. All patients diagnosed with an invasive melanoma involving the cartilaginous ear between 1990 and 2022 were included. Data analysis was performed to assess the association between cartilage resection and local recurrence (LR), recurrence-free survival (RFS), melanoma-specific survival (MSS), and overall survival (OS). Patient files, operation and pathology reports, and follow-up letters were reviewed when any ambiguity was present. Cartilage resection was determined either by the presence of cartilage noted in the histopathology report and/or description of a cartilage resection in the operation report. When available, slides of patients with reported invasion into cartilage or perichondrium were reviewed by an expert dermatopathologist for confirmation. Patients with in situ disease only or tumors located at the earlobe were excluded.

The follow-up period was calculated from the date of definitive treatment of the primary melanoma to the last follow-up visit or death. Data were summarized using frequencies and proportions for categorical variables and means (with standard deviations) and medians (with ranges) for continuous variables.

Patient characteristics and clinicopathological features were summarized and stratified by cartilage resection (categorized as cartilage resection or cartilage sparing). RFS, MSS, and OS were analyzed using the Kaplan–Meier method stratified by cartilage resection. Landmark survival rates at 3 and 5 years were derived along with their 95% confidence intervals (CIs). Survival difference between groups was evaluated using the log-rank tests. RFS time was defined as the time from the date of definitive surgery to the date of the first recurrence. Patients without recurrence were censored at the date of their last follow-up visit or death. MSS was defined as the time between the date of definitive surgery and the date of death due to melanoma. OS time was defined as the time between the date of definitive surgery and the date of death or last date of follow-up.

Univariable logistical and Cox regression analyses were performed using the Wald test and Cox proportional hazards model to examine for associations between the endpoints of interest and primary melanoma pathology details, anatomical site, age, and American Joint Committee on Cancer (AJCC) staging. Cartilage-resected and cartilage-spared patients were matched on multivariable analysis with respect to age, Breslow thickness, ulceration, mitotic rate, and anatomical site. All statistical analyses were performed using SAS 9.4 (SAS Institute Inc. Cary, NC, USA) and R version 4.1.3 (R Core Team, Vienna, Austria). Statistical significance was determined at a *p*-value <0.05.

This study was approved by the MIA Research Committee (MIA2024/494) and the Sydney Local Health District Ethics Review Committee (X15-0311 and 2019/ETH06854). All patients signed informed consent for inclusion of their data in the institutional database.

## Results

**Fig. 1:**
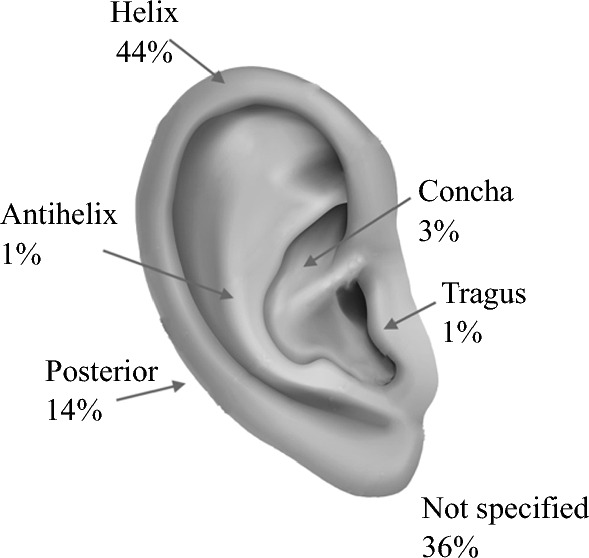
Distribution of location of the primary tumor

The baseline characteristics and pathological features of this patient cohort are summarized in Table 1. Overall, 411 patients had a median (range) age of 65 years (6–97), including 312 (76%) male patients. Distribution was similar between the right (47%) and left (53%) ears. The most common tumor location was the helix (44%) followed by the posterior ear (14%) [Fig. 1]. A greater proportion of tumors located at the helix underwent cartilage resection (47% vs. 30%; *p* ≤ 0.0001), whereas a greater proportion of posterior ear tumors underwent a cartilage-sparing procedure (7% vs. 42%; *p ≤* 0.0001). The median Breslow thickness was 1.3 mm and 9.5% of patients had stage III or IV disease at presentation. The cartilage resection group had a higher mean Breslow thickness (1.9 vs. 1.4 mm; *p* = 0.0002) and stage IIA disease (16.7 vs. 8.6%; *p* = 0.041), whereas the cartilage-sparing group had a higher proportion of stage IA disease (60.5 vs. 39.7%; *p* = 0.041). There were no other statistically significant differences between patient demographics or tumor characteristics between the two groups.

**Table 1:** Summary of patient demographics and pathological features stratified by cartilage surgery

Characteristics	Overall [*N* = 411]	Cartilage resected [*n* = 330]	Cartilage spared [*n* = 81]	*p* Value
Age, years [median (range)]	65 (6–97.4)	65.8 (6–97)	63 (19–91)	0.3677
Gender				
Female	99 (24.1)	81 (24.5)	18 (22.2)	0.6613
Male	312 (75.9)	249 (75.5)	63 (77.8)	
Laterality				
Left	218 (53.0)	169 (51.2)	49 (60.5)	0.1337
Right	193 (47.0)	161 (48.8)	32 (39.5)	
Primary site				
Helix	180 (43.8)	156 (47.3)	24 (29.6)	<0.0001
Antihelix	4 (1.0)	4 (1.2)	0 (0.0)	
Posterior	57 (13.9)	23 (7.0)	34 (42.0)	
Fossa	6 (1.5)	6 (1.8)	0 (0.0)	
Tragus	5 (1.2)	3 (0.9)	2 (2.5)	
Concha	11 (2.7)	9 (2.7)	2 (2.5)	
Ear, NOS	148 (36.0)	129 (39.1)	19 (23.5)	
Primary site (simplified)				
Anterior^a^	206 (50.1)	178 (53.9)	28 (34.6)	<0.0001
Posterior	57 (13.9)	23 (7.0)	34 (42.0)	
Ear, NOS	148 (36.0)	129 (39.1)	19 (23.5)	
Breslow thickness, mm [median (range)]	1.3 (0.2–18.0)	1.5 (0.2–18.0)	0.9 (0.2–7.8)	0.0002
Ulceration				
No	290 (70.6)	231 (70.0)	59 (72.8)	0.0658
Yes	76 (18.5)	67 (20.3)	9 (11.1)	
Unknown	45 (10.9)	32 (9.7)	13 (16.0)	
Mitoses, mm^2^ [median (range)]	2.0 (0–40)	2.0 (0–40)	2.0 (0–28)	0.2247
Histogenesis				
Superficial spreading	170 (41.4)	134 (40.6)	36 (44.4)	0.0771
Desmoplastic	21 (5.1)	20 (6.1)	1 (1.2)	
Nodular	88 (21.4)	75 (22.7)	13 (16.0)	
Lentigo maligna	56 (13.6)	40 (12.1)	16 (19.8)	
Other	7 (1.7)	4 (1.2)	3 (3.7)	
Unknown	69 (16.8)	57 (17.3)	12 (14.8)	
Lymphatic invasion				
Absent	285 (69.3)	224 (67.9)	61 (75.3)	0.4273
Present	7 (1.7)	6 (1.8)	1 (1.2)	
Unknown	119 (29.0)	100 (30.3)	19 (23.5)	
Vascular invasion				
Absent	292 (71.0)	231 (70.0)	61 (75.3)	0.5340
Present	8 (1.9)	6 (1.8)	2 (2.5)	
Unknown	111 (27.0)	93 (28.2)	18 (22.2)	
AJCC stage (8th edition) at primary diagnosis				
IA	180 (43.8)	131 (39.7)	49 (60.5)	0.0410
IB	74 (18.0)	61 (18.5)	13 (16.0)	
IIA	62 (15.1)	55 (16.7)	7 (8.6)	
IIB	38 (9.2)	33 (10.0)	5 (6.2)	
IIC	14 (3.4)	11 (3.3)	3 (3.7)	
IIIA	6 (1.5)	4 (1.2)	2 (2.5)	
IIIB	12 (2.9)	11 (3.3)	1 (1.2)	
IIIC	21 (5.1)	20 (6.1)	1 (1.2)	
Unknown	4 (1.0)	4 (1.2)	0 (0.0)	
SLNB performed				
No	268 (65.2)	208 (63.0)	60 (74.1)	0.0615
Yes	143 (34.8)	122 (37.0)	21 (25.9)	
SLNB positivity				
Negative	118 (82.5)	100 (82.0)	18 (85.7)	0.6763
Positive	25 (17.5)	22 (18.0)	3 (14.3)	
Recurrence type				
No recurrence	320 (77.9)	256 (77.6)	64 (79.0)	0.6348
Local	22 (5.4)	19 (5.8)	3 (3.7)	
In transit	12 (2.9)	11 (3.3)	1 (1.2)	
Regional	36 (8.8)	29 (8.8)	7 (8.6)	
Distant	21 (5.1)	15 (4.5)	6 (7.4)	

Data are expressed as *n* (%) unless otherwise specified

*NOS* not otherwise specified, *AJCC* American Joint Committee on Cancer, *SLNB* sentinel lymph node biopsy

^a^Anterior – Helix, Antihelix, Fossa, Tragus, Concha

**Fig. 2:**
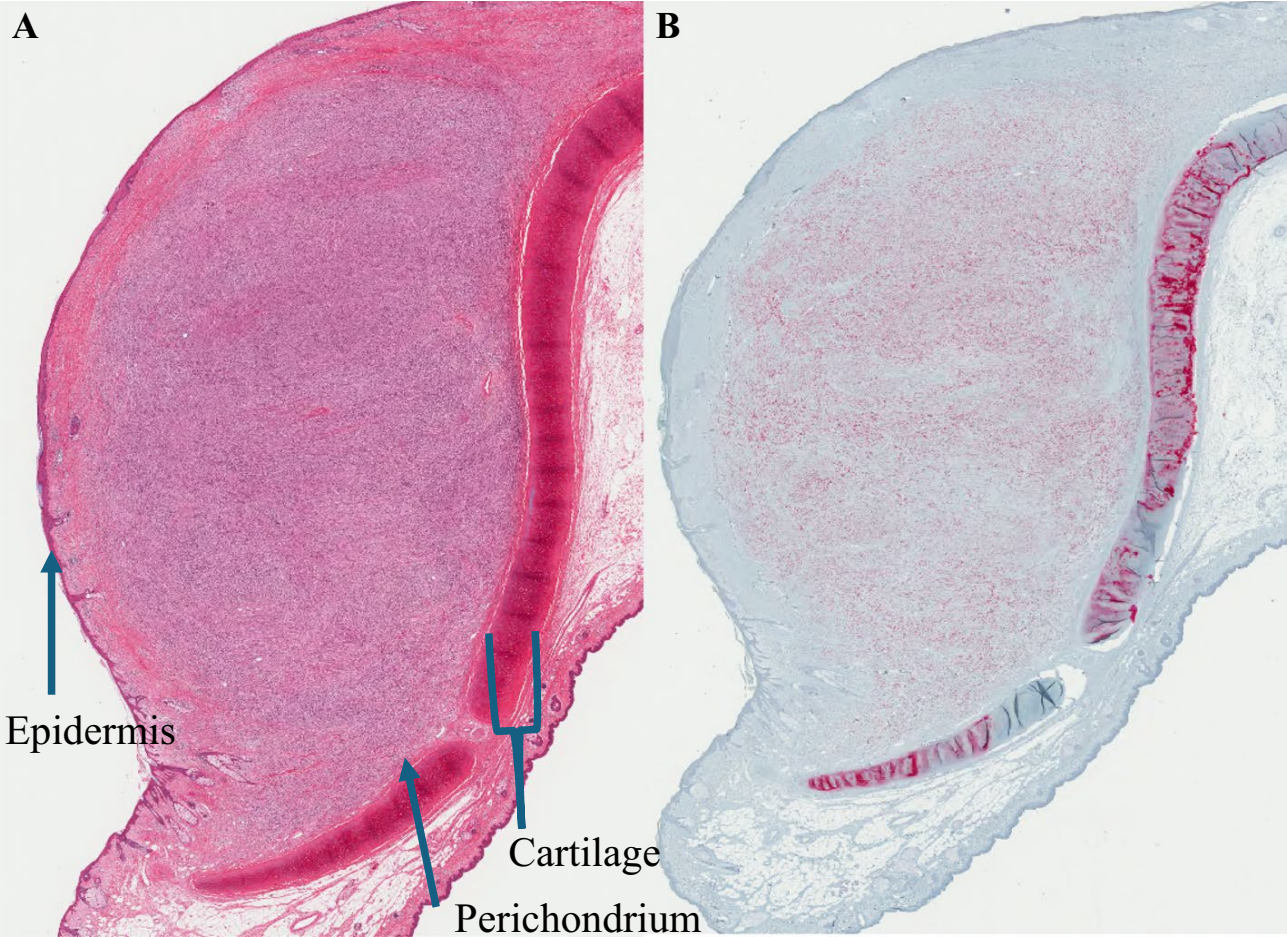
(**A**) Hematoxylin and eosin. (**B**) SOX10 immunohistochemical section of resected ear melanoma demonstrating invasive melanoma abutting perichondrium but not invading into cartilage

A total of 330 patients (80%) underwent cartilage resection, with 81 (20%) having a cartilage-sparing procedure. Of those patients who had cartilage resected, none demonstrated cartilage invasion and 6 (1.8%) had tumor abutting perichondrium but not invading beyond (Fig. 2). Factors associated with an increased likelihood of cartilage resection included Breslow thickness 2.01–4.0 mm {hazard ratio (HR) 3.40 [CI 1.05–11.06]} on multivariate analysis and patients with AJCC 8th Edition stage IIA disease (HR 2.94 [CI 1.25–6.89]) [Table 2]. Sentinel lymph node biopsy (SLNB) was performed in 143 (35%) patients, with 17% returning a positive result.

**Table 2:** Univariate and multivariate logistic regression model for cartilage surgery (1 = resected, 0 = spared)

Variable	Univariable		Multivariable	
	OR (95% CI)	*p* Value	OR (95% CI)	*p *Value
Age (categorized)				
≤65	1	0.2758	1	0.3079
>65	1.31 (0.81–2.14)		1.35 (0.76–2.42)	
Ulceration				
No	1	0.0722	1	0.2164
Yes	1.90 (0.90–4.03)		1.56 (0.58–4.17)	
Unknown	0.63 (0.31–1.27)		0.51 (0.20–1.27)	
Breslow (categorized)				
≤1	1	0.0121	1	0.2294
1.1–2.0	1.96 (1.07–3.57)		1.56 (0.74–3.29)	
2.1–4.0	3.84 (1.66–8.92)		3.70 (1.16–11.79)	
>4	1.96 (0.77–4.97)		3.08 (0.84–11.28)	
Unknown	48430 (0.00–3E180)		26320 (0.00–7E174)	
Mitoses (categorized)				
0	1	0.2250	1	0.1598
1–2	1.60 (0.79–3.23)		2.01 (0.84–4.79)	
3–4	0.97 (0.44–2.16)		0.54 (0.20–1.47)	
≥5	1.79 (0.90–3.57)		0.81 (0.31–2.16)	
Unknown	0.82 (0.37–1.84)		1.09 (0.39–3.03)	
Primary location (simplified)				
Anterior	1	<0.0001	1	<0.0001
Posterior	0.11 (0.05–0.21)		0.08 (0.04–0.17)	
Ear, NOS	1.07 (0.57–2.00)		1.14 (0.60–2.16)	

*OR* odds ratio, *CI* confidence interval, *NOS* not otherwise specified

Cartilage resection was not associated with improved RFS (HR 0.82 [0.52–1.29]; *p* = 0.39), MSS (HR 0.89 [0.30–2.62]; *p* = 0.83), or OS (HR 0.53 [0.28–1.00]; *p* = 0.05) on univariate analysis. Likewise, on multivariate analysis, there was no association between cartilage resection and RFS, MSS, or OS (Tables 3, 4 and 5). Also on multivariate analysis, tumors located at the posterior ear and tragus were associated with improved RFS compared with those located at the helix (HR 13.57 [2.43–75.71] and 8.19 [1.60–41.78], respectively; *p* = 0.002). Twenty-two (5.4%) patients developed LR, with 19 (5.8%) in the cartilage-resected group and 3 (3.7%) in the cartilage-spared group (*p* = 0.63) [Tables 3, 4 and 5].

**Table 3:** Univariate and multivariate Cox regression model for overall survival

Variable	Univariable		Multivariable	
	HR	*p* Value	HR	*p* Value
Cartilage resected				
Spared	1	0.0504	1	0.1246
Resected	0.53 (0.28–1.00)		0.55 (0.26–1.18)	

*HR* hazard ratio

**Table 4:** Univariate and multivariate Cox regression model for recurrence-free survival

Variable	Univariable		Multivariable	
	HR	*p* Value	HR	*p* Value
Cartilage resected				
Spared	1	0.3901	1	0.5202
Resected	0.82 (0.52–1.29)		0.76 (0.32–1.77)	

*HR* hazard ratio

**Table 5:** Univariate and multivariate Cox regression model for melanoma-specific survival

Variable	Univariable	
	HR	*p* Value
Cartilage resected		
Spared	1	0.8310
Resected	0.89 (0.30–2.62)	

*HR* hazard ratio

**Fig. 3:**
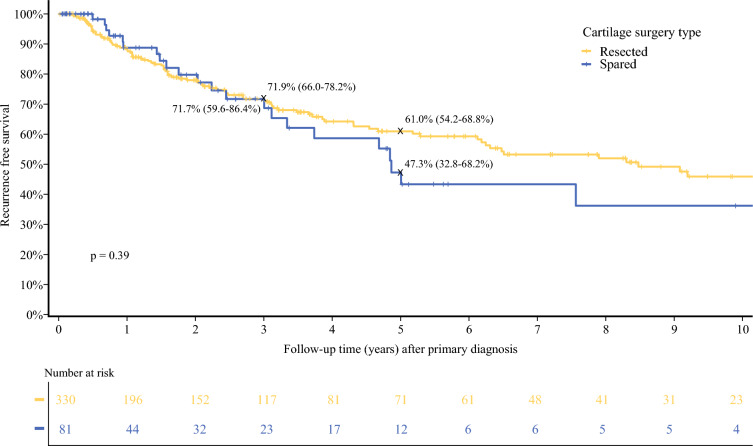
Recurrence-free survival after primary diagnosis by cartilage surgery type

**Fig. 4:**
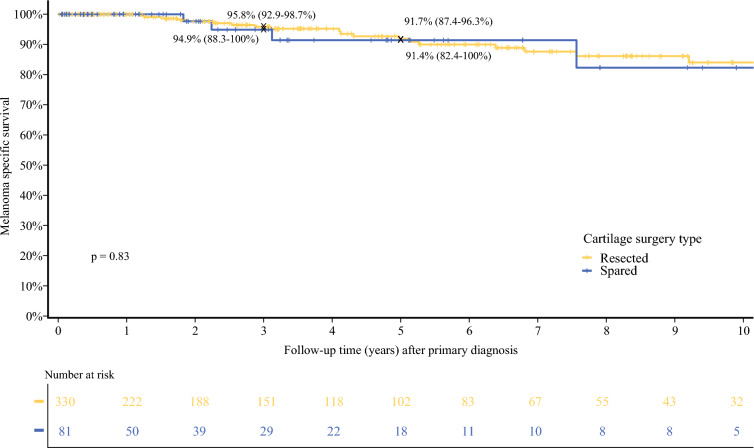
Melanoma-specific survival after primary diagnosis by cartilage surgery type

**Fig. 5:**
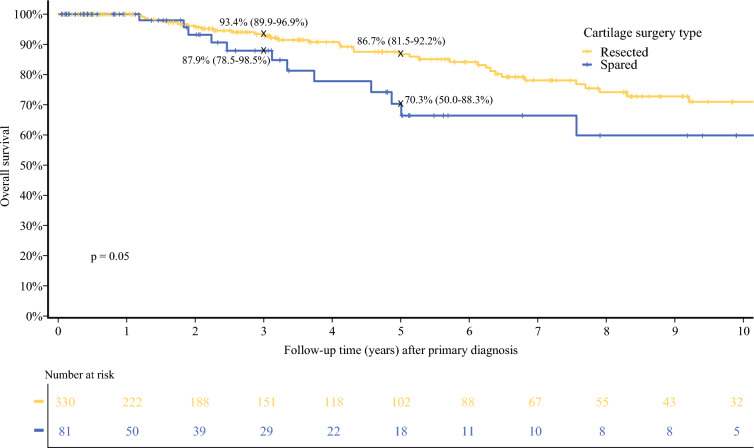
Overall survival after primary diagnosis by cartilage surgery type

Five-year RFS was 61% (54.2–68.8) in the cartilage resection group versus 47.3% (32.8–68.2) in the cartilage-sparing group (*p* = 0.39) [Fig. 3]. Five-year MSS was 91.7% (87.4–96.3) versus 91.4% (82.4–100) in the two groups (*p* = 0.83) [Fig. 4]. Five-year OS survival was 86.7% (81.5–92.2) for patients who had cartilage resected versus 70.3% (50–88.3) in patients who had cartilage spared (*p* = 0.05) [Fig. 5]. Median follow-up time was 3.1 years (IQR 0.86–7.75), with a median time to recurrence of 1.88 years (IQR 0.52–4.58).

## Discussion

This study represents the largest cohort to date comparing melanoma-specific outcomes for patients having cartilage-sparing surgery of the ear with those who have cartilage resected for management of invasive melanoma. In this cohort, cartilage resection did not confer an MSS advantage and did not result in lower recurrence rates. This study suggests that the decision to perform a cartilage-sparing procedure is unlikely to compromise oncological safety and could be considered if the resulting reconstruction will provide an improved functional and cosmetic outcome to the patient.

The largest cohort to date prior to this study was by Truong et al.^11^, which described 156 patients with external ear melanomas, of whom 18.6% had a cartilage-sparing operation. In their cohort, there was no RFS advantage for patients who had cartilage resection, with a 5-year RFS and OS of 84.5 and 79.0% respectively, and a median follow-up of 5.5 months. However, there were several limitations of this study, including the inclusion of melanomas of the non-cartilaginous earlobe and the definition of cartilage-sparing surgery as any operation that was not described as a wedge resection in the procedure report, which is likely to overestimate the number of cartilage-sparing procedures. In our study, we defined a cartilage-sparing procedure as one where cartilage was not identified in the histological analysis, as well as the lack of description of a cartilage resection in the procedure report, which we believe to be a more accurate reflection of the true number of cartilage-sparing procedures. Likewise, in the retrospective review by Truong et al. only early-stage melanomas were included, with a median Breslow thickness of 0.86 mm, which represents a majority of patients who would ordinarily have a lower risk of recurrence and melanoma-specific mortality. The present study had a greater median Breslow thickness of 1.3 mm and 9.5% of patients had stage III disease, which represents a cohort of more advanced-stage disease than these previous studies.

To date, there have been 11 studies describing a cartilage-sparing approach to the surgical management of external ear melanoma, as summarized in Table 6.^3–13^ While only small retrospective series and case studies, they all demonstrate acceptable RFS and MSS outcomes. Of the six studies that compared cartilage resection with cartilage-sparing surgery, none reported invasion of melanoma into the cartilage.^3–5,7,9,11^ This mirrors the findings in the present study in which none of the 330 patients who had cartilage resected had evidence of melanoma invasion on histopathology, and only 6 demonstrated tumor abutting perichondrium. In these 6 patients, all but one had T4a primary lesions, which could suggest that thicker primaries may still benefit from cartilage resection. However, in this cohort, 39 patients with T4a or T4b lesions had cartilage resected, with 34 (87%) not abutting perichondrium. Therefore, despite a small number probably needing cartilage resection, the majority will achieve clear margins even for thicker primary tumors. To our knowledge, there has only been one published report of invasion into cartilage.^13^ In that study, Benmeir et al. report a cohort of thick melanomas in which 8/13 patients demonstrated invasion into the cartilage. Our findings, together with the majority of similar published literature, refutes this finding and suggests that the overwhelming pattern of local invasion of melanoma of the ear is a peripheral pattern along fascial planes, superficial to the cartilage, rather than penetrating deep to perichondrium.

**Table 6:** Previously published studies comparing cartilage resection with cartilage-sparing procedures

First author	Year	Country	*N*	No. of cartilage-sparing procedures	Mean Breslow thickness (mm)	MSS (%)	RFS (%)	Cartilage involvement	Median follow-up (months)
Yamasaki^5^	2020	USA	8	8	1.2	100	87.5	No	22
Craig^6^	2012	USA	51	2	1.6	NA	NA	No	NA
Harrison^7^	2019	UK	40	29	2.1	94	85	No	NA
McCarty^8^	2013	USA	18	18	2.0	89	100	No	30
Cole^3^	1992	USA	31	3	1.7	71	67.7	NA	84
Pockaj^4^	2003	USA	78	7	1.7	77	76	NA	84
Sartore^9^	2012	Italy	9	2	2.1	89	NA	No	72
Thuile^10^	2018	Italy	1	1	1.3	100	100	NA	44
Truong^11^	2020	USA	156	14	0.9	95.5	84.5	NA	66
Cohen^12^	1990	USA	1	1	4.3	NA	NA	No	NA
Benmeir^13^	1995	Israel	13	2	3.7	31	NA	Yes	NA

*NA* not available, *MSS* melanoma-specific survival, *RFS* recurrence-free survival

The decision to resect cartilage is not solely based on oncological negative margins but should also consider patient factors such as skin quality and potential inhibitors of wound healing, such as smoking, diabetes, and immunosuppression, which may inhibit the healing of skin grafts and local flaps. Functional aspects should also be considered, including the need to wear glasses or hearing aids. This study demonstrates that a cartilage resection is not essential to achieve good oncological outcomes for patients with invasive melanomas, even if presenting with high-risk primary lesions.

Limitations of this study include the retrospective design, which resulted in some missing data points, including 36% of patients who did not have the exact location of their lesions described in the database, clinical notes, or histological report. However, we hypothesize that the proportion of patients in each anatomical site would remain relatively similar and in keeping with the distribution of tumors described in other studies, with the helix being the most common site of occurrence.

Despite this, we report a large cohort of a relatively uncommon melanoma location. To our knowledge, this was the first study to compare melanoma-specific outcomes between patients who have cartilage resection and those who have a cartilage-sparing procedure. In addition, in this large cohort of ear melanomas, we confirm the findings of previous studies that have demonstrated no invasion beyond the perichondrium. The assumption is that cartilage-sparing procedures will lead to a more cosmetically and functionally favorable outcome. Future prospective trials including patient-reported outcomes and quality-of-life measures in these two groups would be helpful.

## Conclusion

Cartilage resection in the surgical management of invasive melanoma of the ear does not appear to alter RFS, MSS, or OS compared with patients who have a cartilage-sparing procedure. Particularly in early-stage disease, the decision to perform a cartilage-sparing procedure should be made on an individual basis, considering patient factors, cosmesis, and functional outcomes, rather than concern for adequate oncological clearance, as melanoma does not appear to invade beyond the perichondrium.
